# Bursts of Vertex Activation and Epidemics in Evolving Networks

**DOI:** 10.1371/journal.pcbi.1002974

**Published:** 2013-03-21

**Authors:** Luis E. C. Rocha, Vincent D. Blondel

**Affiliations:** Department of Mathematical Engineering, Université catholique de Louvain, Louvain-la-Neuve, Belgium; Pennsylvania State University, United States of America

## Abstract

The dynamic nature of contact patterns creates diverse temporal structures. In particular, empirical studies have shown that contact patterns follow heterogeneous inter-event time intervals, meaning that periods of high activity are followed by long periods of inactivity. To investigate the impact of these heterogeneities in the spread of infection from a theoretical perspective, we propose a stochastic model to generate temporal networks where vertices make instantaneous contacts following heterogeneous inter-event intervals, and may leave and enter the system. We study how these properties affect the prevalence of an infection and estimate 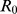, the number of secondary infections of an infectious individual in a completely susceptible population, by modeling simulated infections (SI and SIR) that co-evolve with the network structure. We find that heterogeneous contact patterns cause earlier and larger epidemics in the SIR model in comparison to homogeneous scenarios for a vast range of parameter values, while smaller epidemics may happen in some combinations of parameters. In the case of SI and heterogeneous patterns, the epidemics develop faster in the earlier stages followed by a slowdown in the asymptotic limit. For increasing vertex turnover rates, heterogeneous patterns generally cause higher prevalence in comparison to homogeneous scenarios with the same average inter-event interval. We find that 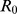 is generally higher for heterogeneous patterns, except for sufficiently large infection duration and transmission probability.

## Author Summary

Networks of sexual contacts and of spatial proximity are of interest for the understanding of epidemics because they define potential pathways by which sexual and airborne infections spread. These networks are not static but vary, with both vertices and links appearing and disappearing at different times. One of the temporal properties observed across systems is that the time lapse between two contacts is irregular, which means that high activity is followed by long intervals of idleness. In this article, by using a theoretical model of a dynamic network co-evolving with a simulated infection, we show that such heterogeneity leads to earlier epidemic outbreaks and increased prevalence of infections for a range of parameters, in comparison to scenarios of regular activity, which is the current modeling paradigm in mathematical epidemiology. We also include a turnover rate to model individuals entering and leaving the system, and we show that if turnover is high, the relative difference in the prevalence of heterogeneous and homogeneous contact patterns increases due to the continuous influx of susceptible individuals. These heterogeneities also increase the expected number of secondary infections produced by a single infected vertex in a completely susceptible population.

## Introduction

Living in society implies that individuals are constantly interacting with each other. Interactions may take different forms, but those involving proximity or direct contact are of special interest to epidemiology because they are potential bridges by which infections may propagate. These interactions can be modeled by using the formalism of networks, where a vertex represents a person and the interaction between two persons corresponds to a link [1], [2]. Research on empirical data indicates that contact patterns are not random but contain network structures whose particularities depend on the environment and context of the contacts [1], [2]. These interactions have in common the property of being highly heterogeneous in the sense that each individual interacts with a different number of other people, belongs to a different community, or assumes different topological roles in the network. Several studies using networks have assumed that contacts are fixed, or static, and the topology at different levels contains the relevant structures to regulate the spreading processes [3]–[7]. The static approach is typically used as a first approximation to contact patterns due to its simplicity, but it is more suitable for modeling systems where processes propagate faster than the evolution of the network. Nevertheless, in several networks of interest to epidemiology, the structure may change faster than infectious states, and the static representation becomes deficient [8]. The increasing availability of high-resolution data has shown that contact patterns contain diverse temporal properties [9]–[12] that may affect dynamic processes as much as the topology does. In this new framework, the time and duration during which vertices and links are active are taken into account to more precisely map the contact patterns into an evolving network structure [13]. Some empirical studies show, for example, that contact (or partnership) duration and inter-contact interval (or inter-event time) are highly heterogeneous, which means that the length of partnership do not have characteristic values [11], [12] and intense activity (sometimes called bursts) may be followed by longer intervals of isolation or inactivity [10], [12], [14], . This last property makes it difficult to distinguish from the empirical data whether an individual is absent for some period or simply left the system (i.e., censoring [16]).

Although there is an extensive literature on mathematical models in epidemiology that includes temporal information such as seasonal effects [17], [18], or age-structured populations [18], few studies have addressed the interplay between temporal structures and epidemics [19]. Volz and Meyers, for instance, have studied epidemics in a family of random networks assuming that partners change at a fixed mixing rate and show the importance of contact dynamics to estimate an outbreak of syphilis in a high school [20]. This model is further extended to include heterogeneous contact rates and is applied to study intervention strategies [21]. Smieszek and collaborators have investigated the impact on epidemics of daily changes of partners (equivalent to Volz's model for mixing rate equal to one) and repeated contacts in random networks (equivalent to mixing rate equal to zero), and they discussed the epidemiological contexts where one or another model is more suitable [22]. Another mathematical model that allows detailed tracing of contact behavior and demographic process is used to study the contribution of different infection stages to HIV epidemics [23]. The gap length between different partnerships and the partnership duration have been addressed by different authors, and it has been suggested that these are critical components in sustaining the transmission of gonorrhea [24] and chlamydia [25]. Numerical studies using networks of contact patterns between conference attendees conclude that heterogeneity in contact durations causes lower spread of infection [26]. Furthermore, concurrency has been long claimed, from a theoretical perspective, to be responsible for increasing the prevalence and growth rate of HIV [27].

In this paper, we focus on the heterogeneity in the time interval between two events of vertex activation (hereafter referred to as inter-event time). It has been suggested that, in case of burst activity (e.g., power-law inter-event time distribution), the number of new infected individuals in SI dynamics decays as a power-law in the long-time limit irrespective of the degree distribution of the network [28], [29]. These studies are essentially concerned with the long-term effect of spreading and not with the early stages of an epidemic outbreak. A study using an empirical network of sexual contacts suggests that, in comparison to homogeneous contact patterns, heterogeneous contacts speed up the spread of simulated infections [30]. The results for sexual contacts (together with other similar studies using diverse communication patterns as proxies for contact networks and epidemic–like simulations [31]–[33]) are derived by contrasting the evolution of the infection dynamics in the original network with the same dynamics in randomized versions of the temporal network (null models) where the time stamps are reshuffled retaining some (e.g., daily patterns) or no temporal constraints. Therefore, the relative difference and importance of the temporal characteristics to shape the spread of infections depend on the randomization protocol, and conclusions may be misleading as a result. One example is the increase in average inter-event time caused by the randomization of the time stamps when the turnover of vertices is high. In this case, those vertices that were originally active, for example, only at the beginning of the network, can be found at any time after randomization. Moreover, if samples of the empirical network are subsequently repeated to extend the original network in the time domain, this asymmetry creates artificial cyclic effects since the identity of vertices in the final and initial parts of the network are substantially different.

To avoid conclusions based on particular samples of empirical networks and limitations in reshuffling methods, one can study the spread of infections in a minimalist model containing only the temporal properties of interest. Several assumptions are necessarily made to reduce the complexity of the model, but by using generative models of dynamic networks one can control the temporal structure in a continuous and systematic way. We therefore propose a simple and intuitive theoretical model of a temporal network in which the vertices are active (“on” state) only at certain times and are otherwise inactive (“off” state), co-evolving with an infection dynamic. We mainly focus on susceptible-infective-recovered (SIR) epidemics, but also present results of the susceptible-infective (SI) model, which has typically been used in studies of heterogeneous inter-event times.

## Methods

### Temporal network model

A temporal network may, in its simplest form, be defined as a dynamic network where the vertices and the links are active (“on” state) only at certain times and inactive (“off” state) otherwise [13]. In our model, each vertex follows a stochastic process where subsequent “on” states depend on a certain inter-event time distribution 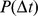. In other words, we have a process in which the probability of a vertex's being active at time 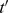 depends on the last time 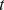 it was active, that is, 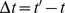. For computational reasons, time is discrete 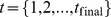 and we generate the next “on” state when the vertex is active, that is, if a vertex is active at 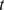, we select the next time it will be active 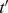 by sampling 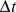 from 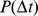 [34]. As soon as a vertex is active, it chooses uniformly between other active vertices, connects to one of them during one time step, and destroys the link afterwards by turning back to “off” state. Such an evolving network is therefore only constrained by the inter-event time and results in randomly mixed networks without degree heterogeneities and correlations. After a few steps everyone has contacted everyone else at least once but since at each time step only one link per vertex is allowed, triads, clicks, or other connected structures do not occur during a single time step. This unrealistic topological structure is necessary to guarantee that only the temporal constraints affect the dynamics. In a related work, Stehlé and co-authors have proposed a dynamic network model able to create various inter-contact times and partnership durations simultaneously. In their model, agents (or vertices) transit between interacting and isolated states, and the transition probabilities depend on both the current state and the time duration since last change of state [35].

The most suitable functional form for the inter-event time distribution depends on the system of interest [10], [12], [14], [15], [31], but for simplicity, we study the two limiting scenarios of burstness (power law) and randomness (Poisson) in our model. As a typical example of a class of skewed distributions we use a power law with an exponential cutoff (or quenched) 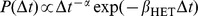 (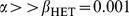), hereafter referred to as HET case. It is suggested that this type of inter-event time distribution appears, as a result of preferential queuing models, for example, where individuals choose to perform highest priority tasks more often than random tasks [14] or because of nonhomogeneous Poissonian processes constrained by cyclic activity [15]. As a baseline for comparison, we consider the exponential distribution 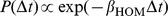 with the same average 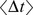 as HET. Exponential inter-event times appear in Poissonian processes where the chance of being active depends only on the constant rate 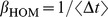 (which means that 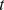 and 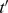 are unrelated) that corresponds to homogeneous inter-event times (HOM). Since our models use discrete time, we use the equivalent geometric distribution in the simulations.

Independently of the evolution of these contacts, we define a vertex turnover mechanism to account for changes in the identity of vertices. For simplicity, we assume a Poissonian process with rate 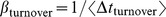 where a new vertex automatically replaces a removed vertex. This procedure keeps the total number of vertices 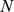 constant (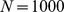) and thus removes the effects of system-size fluctuations. The turnover time of the new vertex is given by 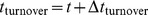 and 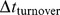 is sampled from the distribution 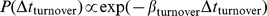. As initial conditions, all vertices start with random values for 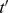 and 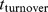. The initial transient quickly disappears after a few time steps and is discarded.

### Epidemics models

On top of the evolving network, we define an infectious process using compartmental models. In these models, vertices belong to compartments such as susceptible (S), infected (I), or recovered (R), according to their state at the moment [18]. Each epidemic model defines a set of possible compartments and allowed transitions between compartments. We focus on general properties of the susceptible-infected-recovered (SIR) dynamics. For comparison with previous studies based on empirical networks, we consider the limit case of susceptible-infected (SI) dynamics. The study of specific infections with realistic parameters and the most proper compartments go beyond the scope of the present article; therefore, we restrict our analysis to theoretical aspects of these standard models to understand the impact of heterogeneous temporal contacts on general spread dynamics.

In the SIR model, a vertex in state I infects a susceptible partner S instantaneously upon contact with probability 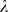 (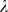 is the per-contact infection probability; unless otherwise stated, as a toy assumption we generally use 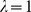). An infected vertex I is completely recovered after 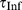 time steps, changing to state R. In contrast, a stochastic recovery parameter would imply that the chance of recovery is the same regardless of the time of infection, which gives relatively large dispersion in the distribution of infective intervals [36]. The SI model simply corresponds to the limit 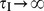. Note that “recover” in the SIR model means that the vertex cannot infect or be infected anymore, but still makes connections until it is removed by the turnover mechanism. Initially, one vertex is set to the infective state I and the remaining 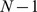 vertices are set susceptible S. Newcomers are always set to susceptible state S. The fraction of infected vertices (or the prevalence) is 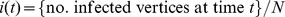; the fractions of susceptible and recovered vertices are defined in the same way and given, respectively, by 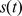 and 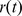. Therefore, we define the outbreak size as 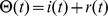. For each simulation, we generate 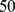 to 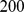 realizations of the random temporal network model (depending on the measure) and, for each realization, we select all vertices active in the first-time step as infection seeds.

## Results

### Evolution of the prevalence

We initially study the deterministic SI infection dynamics in both temporal network models. This model corresponds to an upper limit where transmission occurs upon contact with an infective vertex and a vertex remains infective as time goes on. This simple model, although unrealistic, helps us to understand the essential mechanisms of spreading on the temporal networks and has been also used on related literature.

**Figure 1:**
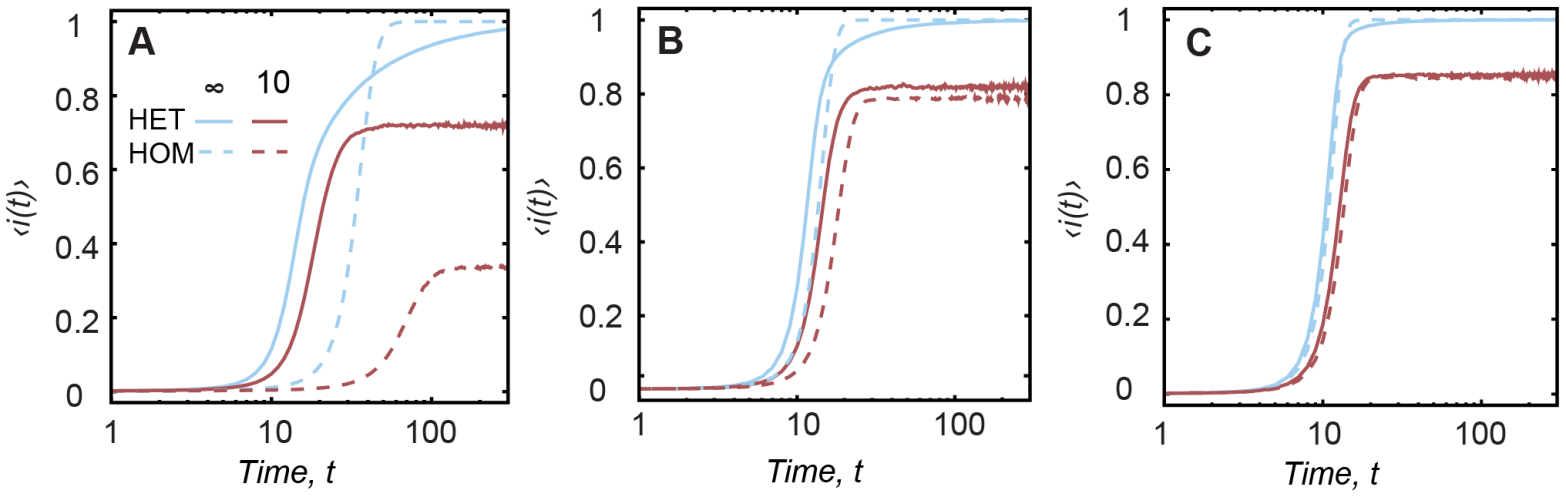
Prevalence of the infection in SI epidemics. The prevalence 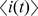 in case of SI epidemics for HET and HOM contact patterns with 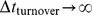 (blue curves) and 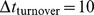 (red curves). Each column corresponds to a different 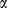, (A) 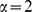, (B) 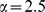 and (C) 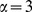. The x-axis is in log-scale.

We see in Figure 1 that in the case of 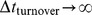 (blue curves), the heterogeneous pattern HET causes a higher prevalence during the initial time interval (e.g., within few time steps, about 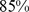 of the population is infected for 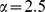 - Figure 1B), followed by a slow increase until the remainder of the network is infected. The homogeneous pattern HOM causes slower growth in the same initial interval; however, it allows the infection to reach the entire network earlier. This initial speedup in the HET occurs because, at short time scales, an infected vertex contacts a larger number of other vertices due to bursts (See Text S1) and thus quickly spreads the infection. As time goes by, the average waiting time to contact a susceptible vertex increases [28], leading to a slowdown in the number of newly infected vertices. The difference between HET and HOM is more pronounced for decreasing 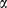 (the slope of the inter-event time distribution) since smaller 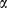 corresponds, in case of HET patterns, to an increasing probability of vertices waiting long intervals between two subsequent activations.

If a moderate turnover is included (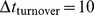 - red curves), HET causes a higher prevalence during the entire period and also removes the asymptotic slow growth as observed in the absence of turnover. This happens because vertices originally inactive for long periods of time are now replaced earlier in the dynamics. The long intervals of inactivity are increasingly removed for increasing turnover rate (decreasing 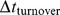). Furthermore, for varying 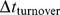 the absence of epidemic outbreak is observed at larger values of 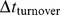 in the HOM case in comparison to HET. This happens because at high turnover rates, vertices following HET patterns make, on average, more contacts before replacement than vertices following HOM, as a consequence, the infection is more likely to die out in the HOM case; in fact, the ratio between the average number of contacts of vertices following HET and HOM increases for decreasing 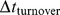 (see Text S1).

**Figure 2:**
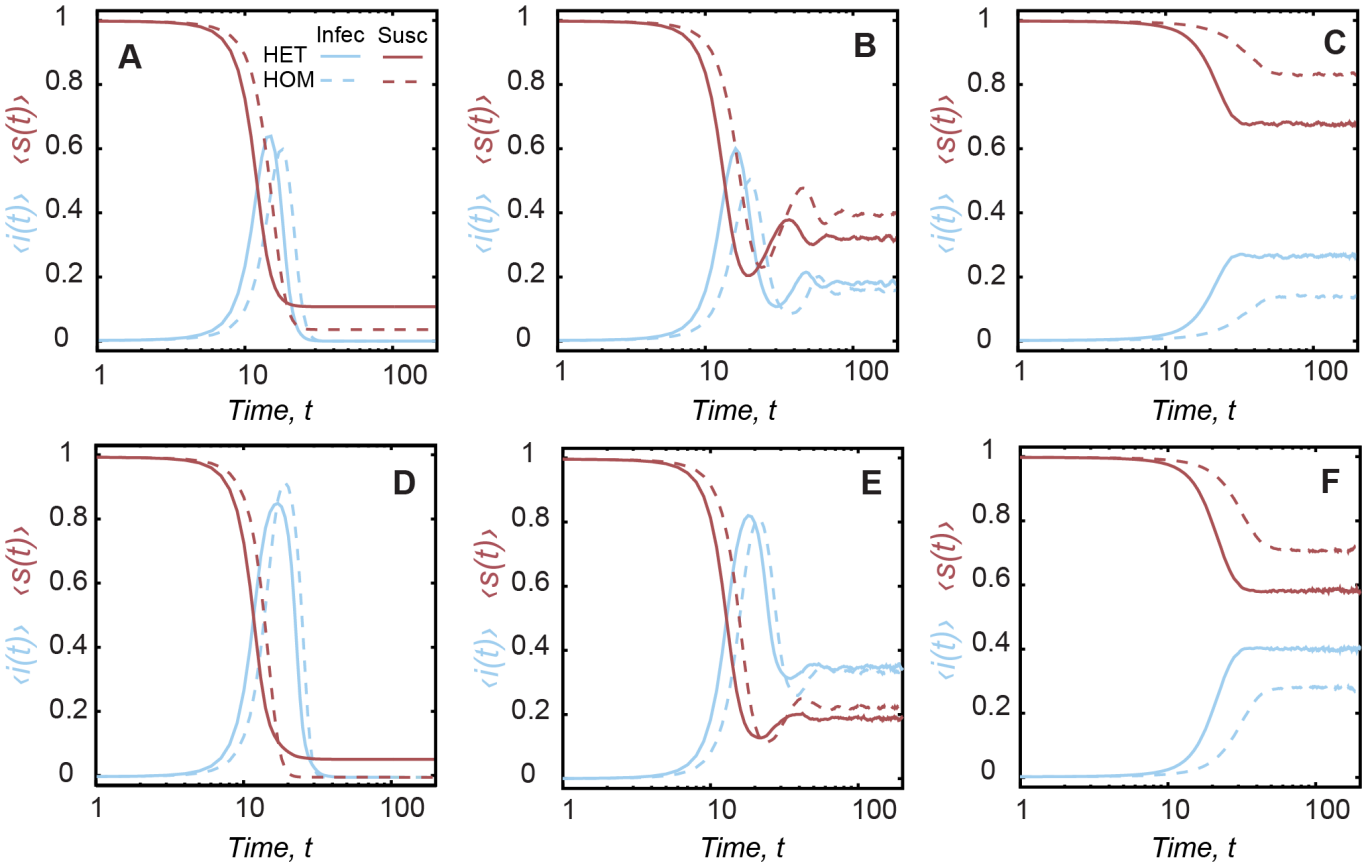
Prevalence of the infection in SIR epidemics. Curves correspond to the fraction of infected 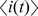 (i.e. the prevalence – blue) and fraction of susceptible individuals 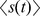 (red). Each panel contains a different configuration: (A)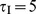 and 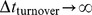; (B) 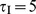 and 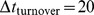; (C) 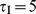 and 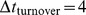; (D) 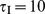 and 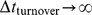; (E) 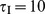 and 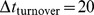; (F) 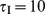 and 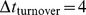. The x-axis is in log-scale.

After discussing the elementary mechanisms of the spread process in this class of temporal networks, we focus in the evolution of the SIR dynamics. The SIR model includes a new compartment where vertices are recovered after a limited period of infectivity. To illustrate the general behavior of the SIR dynamics, we show in Figure 2 the prevalence of the infection within the population for two specific configurations (we explore other configurations bellow) with 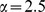 (see Text S1 for the relation between 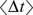 and 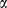) and infective periods 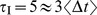 (Figure 2A,B,C) and 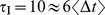 (Figure 2D,E,F). The infection growth has a similar shape for both inter-event time models, but as in the SI dynamics, the curve is shifted depending on the model and parameters; the shorter infective period results on larger difference between the timing of the peak of HET and HOM (Figure 2A). The final fraction of susceptible vertices is higher for HET irrespective of the values of 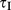 (Figure 2A,D), which suggests that the earlier peak contributes to avoid the infection of all vertices. While in the SI dynamics, the probability that an infected vertex contacts a susceptible vertex decreases after a certain point (due to the long waiting times), in the SIR dynamics, the same vertex recovers before infecting others. As a consequence, vertices inactive for long times remains susceptible. The same mechanism responsible for a non-null number of susceptible vertices also causes the different intensities of the peak prevalence for each network.

The introduction of the turnover mechanism reduces the peak prevalence in both contact patterns but has more impact on the homogeneous network (Figure 2B,C,E,F). As discussed before in the context of SI dynamics, the increasing turnover rate removes the long waiting times and causes HET vertices to have more contacts, at shorter scales, in comparison to HOM vertices (see Text S1). If the infective period is larger, an infected vertex has a higher chance to contact a susceptible vertex and propagate the infection. For moderate turnover, that is, 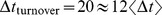 (Figure 2B,E), the newly introduced susceptible vertices are responsible for creating a small second wave of epidemics (peak is 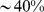 smaller than the first wave for both configurations), followed by a small oscillation of low prevalence for both networks but still slightly higher for heterogeneous contacts. Similar results of the evolution of the epidemics are observed for other configurations with 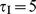 and 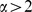 (see Text S1). When the turnover rate is higher (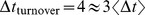), the infection grows monotonically to a state of roughly constant prevalence (Figure 2C,F), leaving a large number of susceptible individuals (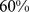 to 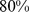 of the vertices for the configurations shown).

### Intensity and timing of peak prevalence

**Figure 3:**
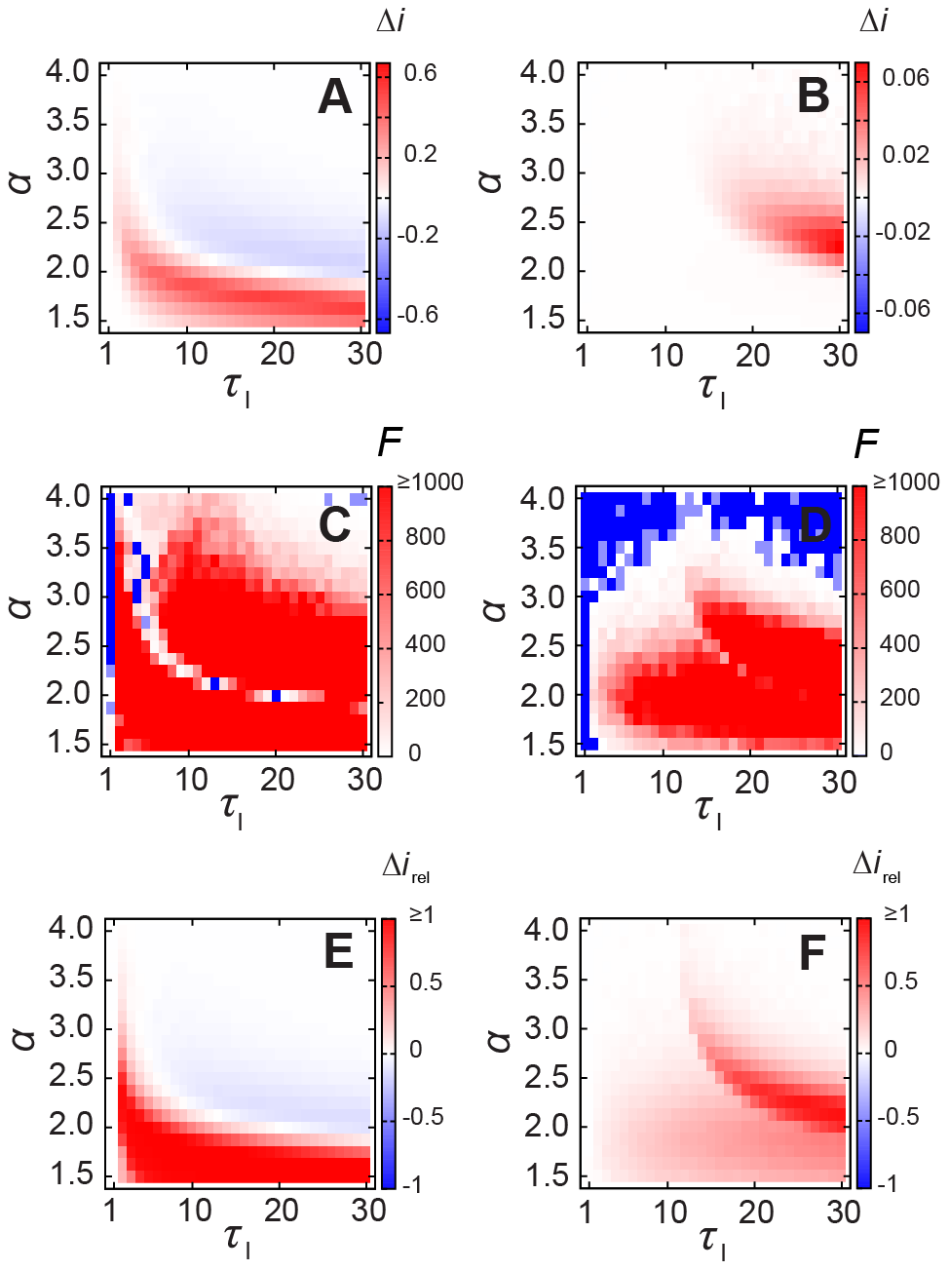
Intensity of the peak prevalence for SIR epidemics. Difference in the intensity of peak prevalence 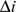 for (A) deterministic (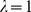) and for (B) stochastic (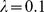) SIR dynamics with various infective intervals in case of 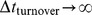. F statistics for (C) deterministic (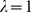) and for (D) stochastic (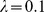) SIR (red and white mean that HET and HOM peak intensities are statistically different, that is, 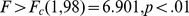), raw p-values are in Text S1; the difference relative to the HET case, that is, 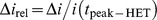 for (E) deterministic (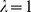) and for (F) stochastic (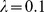) SIR.

In this section we study the dependence of the peak prevalence with the network characteristics and SIR model parameters. When 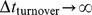, the only temporal pattern is the heterogeneous inter-event times. Figure 3 shows the difference in the intensity of the peak prevalence 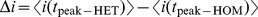 for the two scenarios of inter-event times for the deterministic (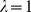, Figure 3A) and stochastic (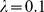, Figure 3B) SIR dynamics. Two distinct regimes appear for difference combinations of the infective interval 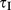 and the contacts heterogeneity (given by the slope 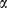 of the power-law distribution). In case of deterministic SIR, for any 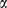 and small values of 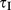, and for 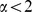 and any 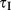, the peak prevalence is higher for heterogeneous contact patterns (positive values of 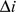). On the other hand, for larger values of the same parameters, we see a higher prevalence in case of the homogeneous contacts HOM (negative values of 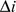). The difference in the peak prevalence between the HET and HOM reaches up to 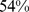 within the range of parameters considered. We perform a simple ANOVA analysis to verify if the intensity of the peak prevalence of the two networks are different and show, through the F statistics for each combination of parameters (Figure 3C), that the differences are statistically significant for a range of values (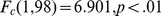, raw p-values are presented in Text S1). We have also calculated the difference 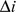 relative to the prevalence in the HET network 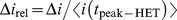. Figure 3E shows that for several configurations, this relative difference goes over 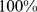, especially for 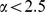. In case of stochastic SIR, we do not observe configurations where the HOM network results on higher prevalence in comparison to the HET model (Figure 3B). The difference 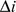 increases for sufficiently large 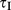 and 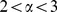. Although the differences in Figure 3B are one order of magnitude smaller than in the deterministic case, they are statistically significant for 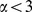 (Figure 3D) and the relative difference remains considerable high, more than 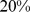 for 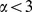 and 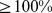 in the dark red region (see Figure 3F).

**Figure 4:**
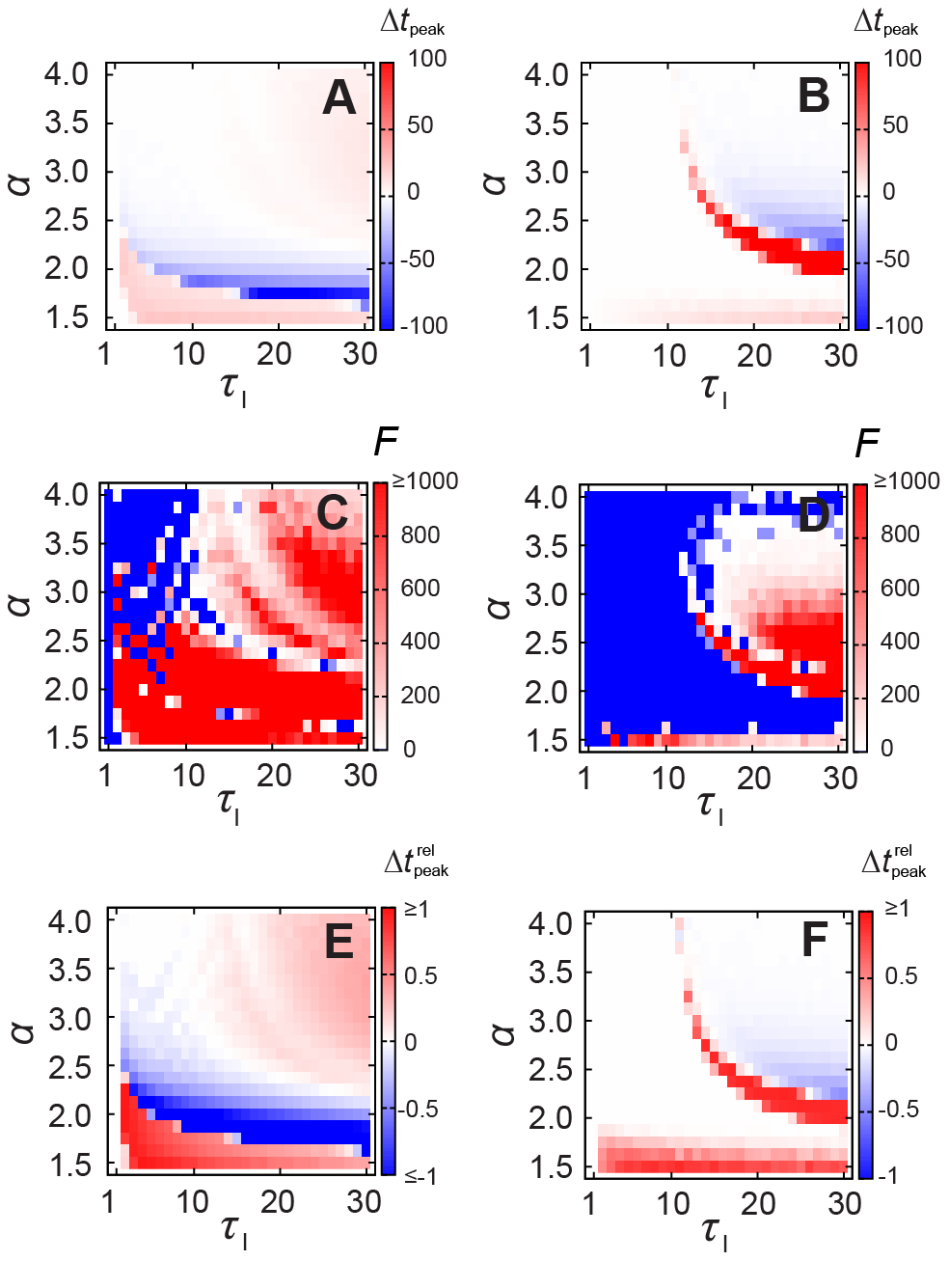
Time of the peak prevalence for SIR epidemics. Difference in the time of peak prevalence 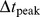 for (A) deterministic (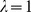) and for (B) stochastic (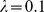) SIR dynamics with various infective intervals in case of 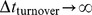; F statistics for (C) deterministic (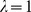) and for (D) stochastic (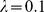) SIR (red and white mean that HET and HOM peak times are statistically different, i.e. 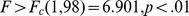), raw p-values are in Text S1; the difference relative to the HET case, that is, 
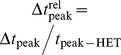
 for (E) deterministic (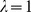) and for (F) stochastic (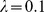) SIR.

The irregular contact patterns also shift the time of the peak prevalence (see Figure 4A,B, where the negative values of 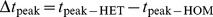 mean that peak HET occurs earlier than peak HOM). In Figure 4A (deterministic SIR), the (positive) region at about 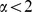 essentially comprises cases in which the infection dies out quickly for homogeneous (and thus 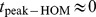) but not for heterogeneous (where 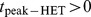) patterns. However, 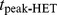 may occur up to 100 time steps earlier than 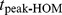 in the interval 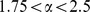 (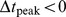). For 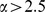 and 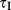 high enough, the epidemic reaches its peak earlier in the HOM scenario; in this regime of sufficiently high 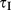 we recover the SI dynamics. The difference between HET and HOM is statistically significant for the red and white region in Figure 4C (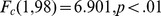; raw p-values are presented in Text S1). The difference relative to the HET case, 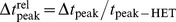, is also large, the blue (red) region in Figure 4E shows that the difference in the time of the peak prevalence can be more than 2 times larger (smaller) than the time of the HET peak prevalence. For stochastic SIR, the difference in the time of peak prevalence (Figure 4B) is statistically significant for 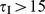 and 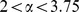 (Figure 4D). Even for stochastic SIR, we identify two different regions in the parameter space where either HET (blue in Figure 4B) or HOM (red in Figure 4B) results on earlier outbreaks. Similarly to the deterministic SIR, the relative differences are consistently high (dark red region in Figure 4F) whenever they are statistically significant.

### Estimation of 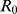


**Figure 5:**
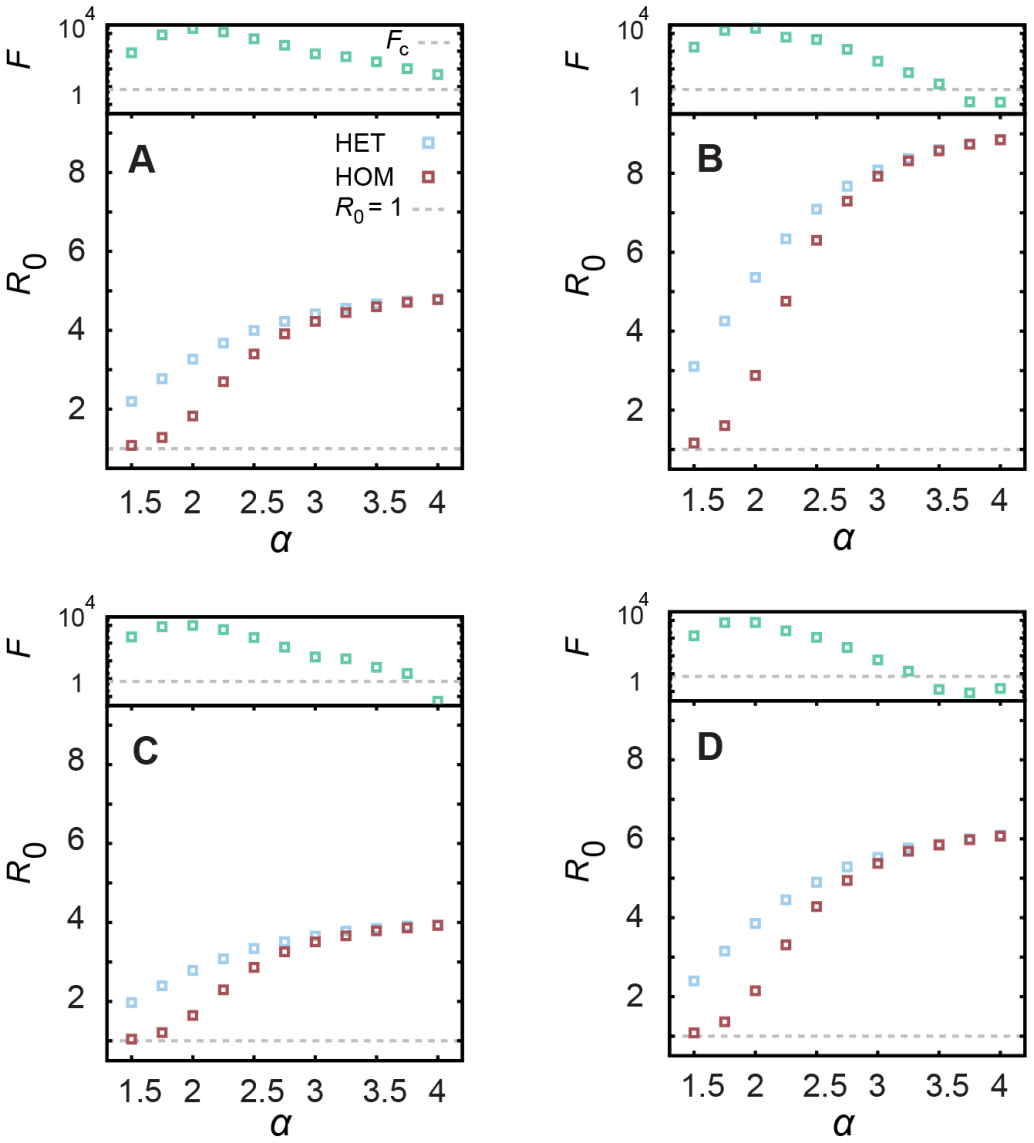
Estimation of 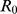 for deterministic SIR epidemics. Numerical estimation of 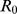 for SIR in case of (A) 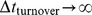 and (C) 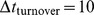 (with 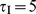), and in case of (B) 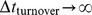 and (D) 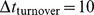 (with 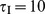). The results are independent of the network size 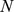 (see Text S1). Dashed lines correspond to 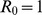. The F statistics is presented above the plots. Dashed lines correspond to 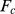; 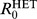 and 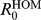 are statistically different if 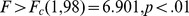.

We estimate 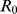 by counting the number of secondary infections of an infectious vertex in a completely susceptible population before the vertex recovers or leaves the system [18]. For the deterministic SIR, Figure 5 shows 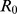 for heterogeneous and homogeneous contact patterns in the case of 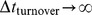 and 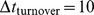 for two different infective intervals 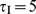 (Figure 5A,C) and 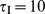 (Figure 5B,D). In all configurations of the homogeneous case, 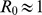 for 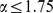. Generally, 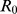 is higher for HET in comparison to HOM. Above the plots, we present the F statistics to quantify the significance of the difference between the two networks and we see that 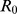 is indistinguishable only in few cases for large 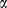. For both infective durations, the values of 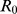 are higher in the case of 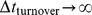 (Figure 5A,B).

**Figure 6:**
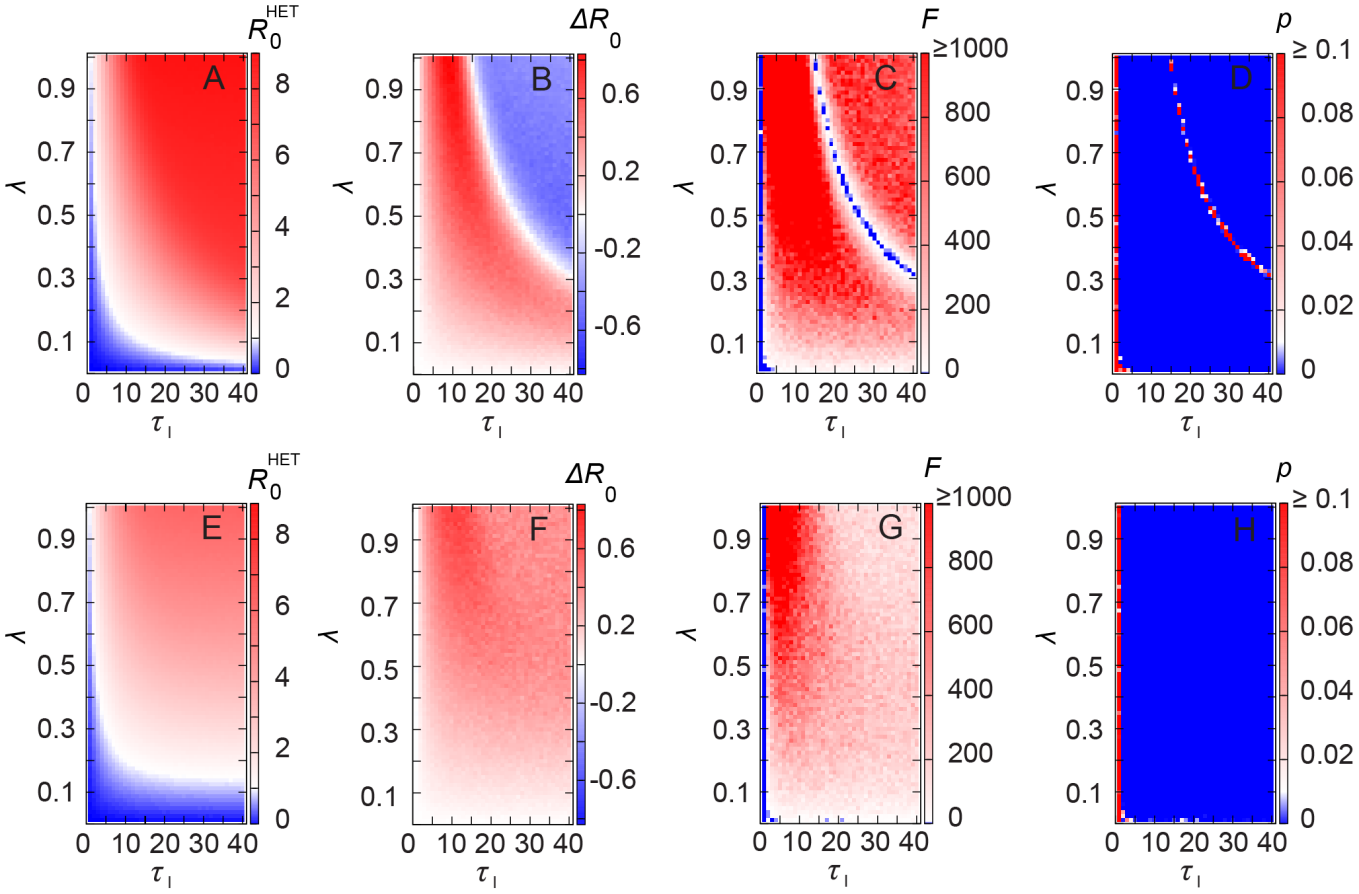
Estimation of 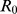 for stochastic SIR epidemics. Numerical estimation of 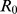 for HET network (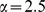) and the difference of 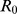 between HET and HOM networks, that is, 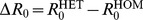. 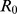 for HET in case of (A) 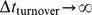 and (E) 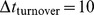; 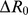 for (B) 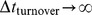 and (F) 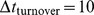; F statistics for (C) 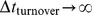 and (G) 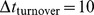 (red and white mean that HET and HOM cases are statistically different, that is, 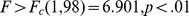); p-values for (D) 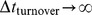 and (H) 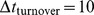.

We show in Figure 6 the effect of stochastic SIR infection dynamics and various infection intervals on 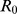 (for 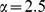 and 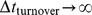). In Figure 6A, we see a threshold (within the numerical accuracy) at 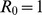 such that epidemics occur above this curve (in the Text S1, we show a similar plot but of the outbreak size, and identify that below this threshold the size of the epidemic outbreak is less than 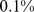). We plot the difference 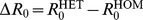 in Figure 6B to facilitate comparison between the two temporal networks. For short infective intervals, HET results in larger 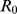 irrespective of the per-contact infection probability 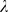. The same applies for small 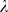 and any infective intervals. On the other hand, the homogeneous contacts result in larger values of 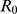 for sufficiently high 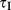 and 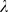. For sufficiently large 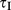, the infection spreads slower in the HET network, as it happens with SI. When the turnover mechanism is included (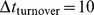), since vertices stay shorter in the dynamics, the absolute values of 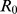 decrease slightly such that 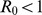 for 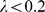 (Figure 6E). Moreover, the heterogeneous network results in larger 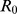 for all studied values (Figure 6F).

### Distribution of outbreaks

**Figure 7:**
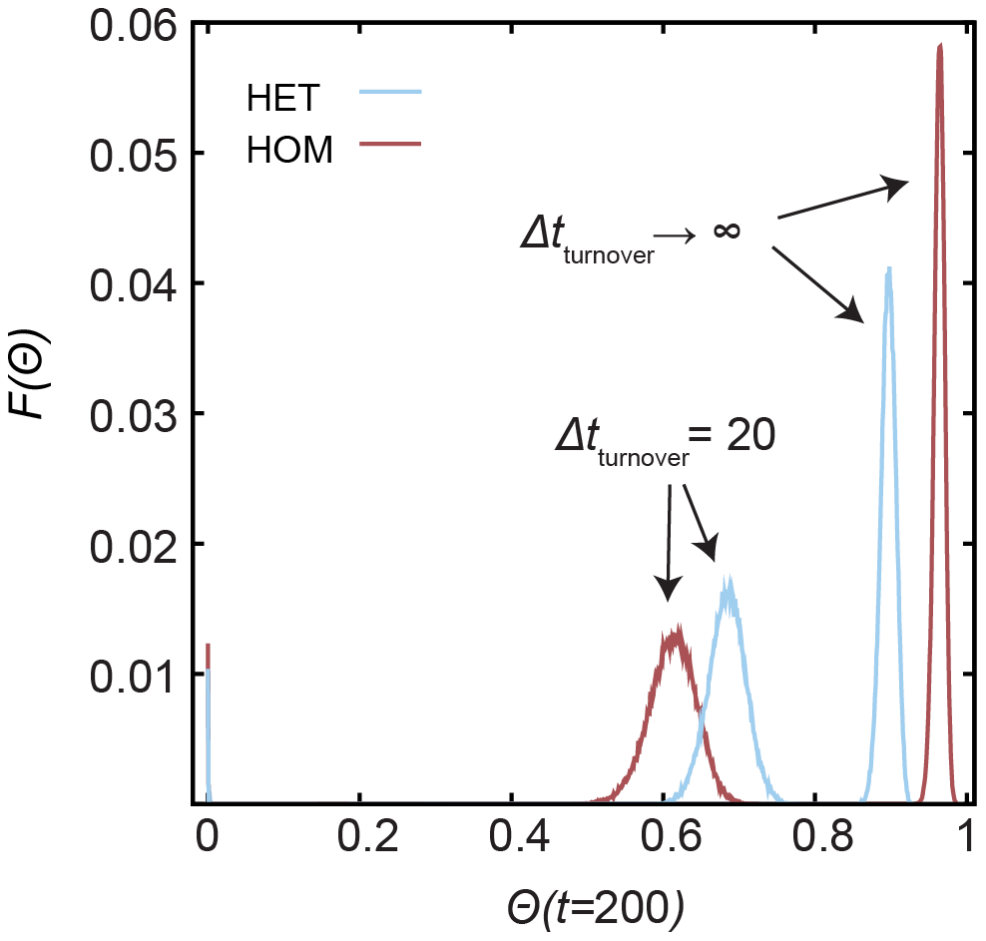
Distribution of outbreak sizes by random initial infection seeds. Fraction of times 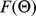 an epidemic outbreak with size 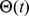 is observed at time 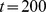. The results correspond to the SIR model with 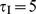 and network configurations with 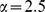.

The introduction of the two temporal constraints in the dynamic network affects in different ways the distribution of possible outbreak sizes for a given initial condition. For 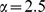, Figure 7 shows that both contact patterns lead to symmetric distributions (of outbreak sizes) with characteristic values but different dispersions. Furthermore, in the case of high turnover rate, a number of initial infections result in null or very small outbreaks since some vertices are removed before infecting others. This effect is more pronounced in the HOM case. This is expected since bursts of activity result in more contacts before the vertex replacement, which is Poissonian (See Text S1). In the absence of turnover, the dispersion of the distribution (measured by the standard deviation) is larger for HET, while in the case of 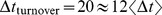, the dispersion is larger for HOM. We measure the standard deviation for some other values of 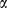 (see Table 1) and observe that, for 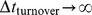, the distribution of outbreaks is broader for the HET in comparison to HOM only for 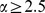 and is narrower for 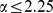. For 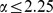, HOM causes several small and null outbreaks and the distribution becomes right skewed, which explains the larger standard deviation. For 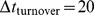, HOM results on larger dispersion than HET for several values of 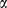; however, in case of 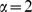, null outbreaks are observed for HOM. In this case of 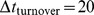, there is also some overlap of the distributions indicating that some initial conditions may give, for example, 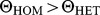, which is different than the characteristic behavior of 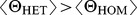.

**Table 1:** The standard deviation of the distribution of outbreak sizes.

	HET	HOM	HET	HOM
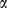	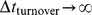		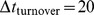	
**2**	0.048	0.069	0.037	0.0003
**2.25**	0.053	0.094	0.028	0.047
**2.5**	0.032	0.020	0.026	0.032
**2.75**	0.016	0.005	0.025	0.026
**3**	0.011	0.002	0.023	0.025

The table contains the standard deviation of the distribution of outbreaks at 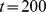 for various values of 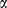, in case of 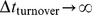 and 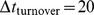. See Figure 7 for the distribution of outbreak sizes for 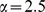. We use 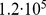 initial infection seeds (SIR dynamics) for each model and combination of parameters.

## Discussion

Contact patterns are characterized by a high degree of heterogeneity both at topological [1], [2] and at temporal levels [9]–[12]. As much as the contact structure (e.g., degree distribution or community structure) constrains the spread of infections, temporal structures (e.g., inter-contact times, causal relations, partnership duration) also influence the dynamics of infection propagation. To contribute to the understanding of the interplay between temporal structures and spread of infections, and in particular, to the role of bursts of vertex activity to regulate epidemics, we introduce a simple and intuitive model of temporal network where the vertex dynamic is constrained only by the inter-event time distribution and by an independent turnover mechanism. Within this simplified framework, the contribution of these temporal constraints to the spread of infections becomes evident since there are no competing topological structures in the model.

Our results show that the prevalence curve can be generally divided into two parts. The first part is characterized by a faster, steeper growth of the fraction of infected vertices in the case of heterogeneous contact patterns. After this initial period, a second regime is identified whose characteristics depend on the epidemics model, turnover rate, and other parameter values. In the absence of replacement of vertices, the prevalence of the infection is generally higher for homogeneous contact patterns. This happens because in a completely susceptible population, an infected vertex quickly contacts several other vertices due to bursts, as a consequence of which the epidemic outbreak occurs earlier. However, as time goes by, the longer inactive intervals due to the broad distribution of inter-event times, the probability of finding a susceptible vertex decreases and the heterogeneous contact patterns slow down the spread of the infection. For some configurations of the SIR dynamics, heterogeneous patterns avoid the infection of the entire network and thus provide a way of decreasing the global impact of the epidemic. Temporal structures may therefore be used to exploit new vaccination protocols based on behavioral characteristics of the population reflected in the dynamic network structures [37]–[39].

In our model, an initial speedup in the SI dynamic is followed by a slowdown in the asymptotic limit, which is in agreement with previous theoretical results [28]–[30]. On the other hand, some other studies (using empirical networks of human communication) suggest that bursts speed up non-deterministic SIR epidemics in the case of small infection probabilities, and slow down for large probabilities [33] and for deterministic SI epidemics [31], [32]. We observe that for decreasing heterogeneity (larger 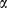), the prevalence is similar for both inter-event time distributions. Therefore, it may be that in networks with skewed (but not strictly power-law such as in Refs. [31], [33]) inter-event time distributions and topological correlations, slower growth eventually occurs in the earlier stages. In other words, while bursts increase the prevalence, at least at shorter time scales, increasing clustering (or community structure) would decrease the spread of an infection. Furthermore, by scanning the parameter space, we observe that homogeneous contacts can cause greater prevalence than heterogeneous contacts for moderate values of infection duration and for large per-contact infection probabilities. It is important to note, however, that we model only one temporal structure. It may be, for example, that heterogeneous partnership durations [26] and non-overlapping partnerships [24], [27] are major temporal structures responsible for slowing spread in empirical networks. Since different structures, both at the temporal and topological level, compete to promote or reduce the spread simultaneously, it remains an open problem to quantify which is the controlling component in this complex dynamic in particular systems.

The increasing of the turnover rate in the dynamics removes the long inter-event times of the vertices (i.e., it is equivalent to a cutoff effect in the power law) since a vertex is more likely to be removed (the replacement is Poissonian in our model) than to wait for very long to be active again. Therefore it is expected that a vertex contacts more different partners before replacement if following heterogeneous inter-event times. Since infected vertices are replaced by susceptible vertices, the increase in turnover causes a faster replacement of infected vertices, leaving more vertices susceptible to infection. Therefore, the difference in prevalence between both contact patterns is more pronounced in networks with high vertex turnover and generally, heterogeneous patterns cause higher prevalence for SI, already for moderate values of vertex turnover. In the case of SIR dynamics, the influx of susceptible vertices causes a second (lower-intensity) wave of infection but the fraction of susceptible vertices remains constant afterwards. Higher rates of vertex turnover cause a larger fraction of the network to remain susceptible in the stationary state.

We have also estimated 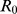, the expected number of secondary infections produced by a single infective vertex in a completely susceptible population. We have found that the difference in 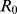 for heterogeneous and homogeneous contact patterns is statistically significant. This relative difference increases with increasing turnover rate. In general, heterogeneous patterns result in higher values of 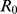, but in case of stochastic SIR dynamics, for sufficiently large values of infection probabilities and duration of infection, homogeneous patterns lead to higher estimates of 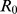. Our results thus indicate that the assumptions of temporally heterogeneous or homogeneous contacts and fixed population give different estimates of 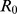 and are therefore relevant to understanding spread in temporal networks.

In summary, we have proposed a simple temporal network model containing heterogeneous time intervals between subsequent vertex activations and a vertex turnover dynamics. The simulation of standard epidemic models (SI and SIR) that co-evolve with this dynamic network has shown that irregular contacts affect significantly the emergence of epidemics. With respect to models that assume characteristic intervals between subsequent vertex activations, the differences in the timing of the epidemic outbreak, the prevalence of the infection, and 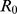 depend not only on the level of temporal heterogeneity of the contacts and vertex turnover, but also on the characteristics and parameters of the epidemics. Further research is needed to understand the contribution of other temporal structures and the combined role of topological and temporal correlations on the emergence of epidemics in dynamic networks.

## Supporting Information

Text S1The text contains information about the values of 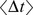 used in the simulations; the ratio of contacts made by a vertex following heterogeneous and homogeneous contact patterns; the effect of varying 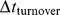 on the SIR dynamics; a finite-size analysis of 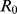 for SIR dynamics; and an analysis of the size of the outbreak for stochastic SIR dynamics.(PDF)
