# Supplementary material for: Bursts of Vertex Activation and Epidemics in Evolving Networks
Source: PLoS Comput Biol. 2013 Mar 21;9(3):e1002974. doi: 10.1371/journal.pcbi.1002974 (PMC3605099; doi:10.1371/journal.pcbi.1002974)
Supplement: Text S1 — The text contains information about the values of used in the simulations; the ratio of contacts made by a vertex following heterogeneous and homogeneous contact patterns; the effect of varying on the SIR dynamics; a finite-size analysis of for SIR dynamics; and an analysis of the size of the outbreak for stochastic SIR dynamics. (PDF) [file pcbi.1002974.s001.pdf]

Supporting Information for:

Bursts of Vertex Activation and Epidemics in Evolving Networks

Luis E C Rocha and Vincent D Blondel

Department of Mathematical Engineering

Université catholique de Louvain, Louvain-la-Neuve, Belgium

Luis.Rocha@uclouvain.be

1. Values of $\langle \Delta t \rangle$ used in the simulations

In the simulations, we use the same $\langle \Delta t \rangle$ for the heterogeneous and homogeneous contact patterns. For a given α, we calculate $\langle \Delta t \rangle$ (Fig. S1) by sampling 10^8 (one hundred million) values from $P(\Delta t) \propto \Delta t^{-\alpha} \exp(-\beta_{\text{HET}} \Delta t)$ (where β_{HET} is fixed and $\alpha \gg \beta_{\text{HET}} = 0.001$). This value of $\langle \Delta t \rangle$ is used in the exponential distribution, $P(\Delta t) \propto \exp(-\beta_{\text{HOM}} \Delta t)$ by setting $\beta_{\text{HOM}} = 1/\langle \Delta t \rangle$. In practice, since we use discrete times, times are sampled from a geometric distribution. Random generators can be found in numerical packages for diverse programming languages, otherwise, see ref. [S1].

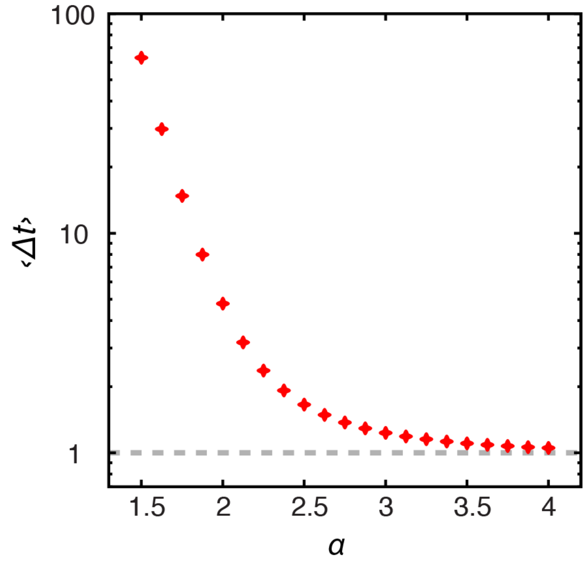

Figure S1: Values of $\langle \Delta t \rangle$ for a given α as obtained by sampling 10^8 values from the power-law (with cutoff) distribution. The y-axis is in log-scale.

2. Number of contacts made by a single vertex

We measure the average number of contacts made by a single vertex, before its replacement, following heterogeneous HET (C_{HET}) and homogeneous HOM (C_{HOM}) contact patterns. We calculate the averages using $5\cdot 10^5$ vertices for each network model. Table S1 shows the ratio between the number of contacts on each case, that is, $C_{\text{HET}}/C_{\text{HOM}}$ for different values of α (columns) and $\Delta t_{\text{turnover}}$ (rows). For decreasing α and $\Delta t_{\text{turnover}}$, this ratio increases, meaning that vertices following heterogeneous patterns make more contacts than vertices following regular contacts at these short time scales. The table also indicates that for sufficiently large α, the number of contacts is equivalent in both network models for any time scale, which explains why the results of the spreading dynamics in the main text are similar for these parameters.

$\Delta t_{\text{turnover}}$ \ α	**1.5**	**2**	**2.5**	**3**	**3.5**	**4**
**4**	17.58	2.61	1.22	1.05	1.01	1.01
**8**	13.89	2.33	1.17	1.04	1.01	1.00
**12**	11.94	2.19	1.14	1.03	1.01	1.00
**16**	10.72	2.08	1.13	1.03	1.01	1.00
**20**	9.83	2.00	1.12	1.02	1.01	1.00
**24**	9.13	1.94	1.10	1.02	1.01	1.00
**28**	8.54	1.91	1.10	1.01	1.00	1.00

Table S1. Ratio $C_{\text{HET}}/C_{\text{HOM}}$ between the number of contacts made by a vertex following heterogeneous C_{HET} and homogeneous C_{HET} patterns.

3. Effect of varying $\Delta t_{\text{turnover}}$ on SIR dynamics

In Figure S2, we fix the infective stage at $\tau_{\text{I}} = 5$ and vary the turnover time $\Delta t_{\text{turnover}}$, for 3 different values of α to show the difference in the prevalence $\Delta i = i_{\text{HET}}(t) - i_{\text{HOM}}(t)$ for the two scenarios of inter-event times during the initial 100 time steps of the epidemics. These results are complementary to Fig. 2 in the main text. For all values of α in the interval $2.25 \le \alpha \le 4$, the pattern of prevalence is similar, with an initial peak for HET, followed by a later peak in case of HOM. For $\alpha \le 2$ and for very high turnover (small $\Delta t_{\text{turnover}}$), heterogeneous HET contact patterns are always higher because they can sustain the epidemics while homogeneous HOM contacts result in multiple null outbreaks. For $\alpha = 2.5$ and for larger values of $\Delta t_{\text{turnover}}$, there is an initial interval where HET prevails, causing higher prevalence, followed by a period where HOM results on higher prevalence. On the other hand, the sign of Δi alternates during the observed 100 time steps for decreasing $\Delta t_{\text{turnover}}$. The second period of higher prevalence for HOM (the second wave) appears earlier for decreasing $\Delta t_{\text{turnover}}$. This behavior is similar for values of $\alpha > 2.5$, but the absolute values of Δi are smaller. On the other hand, for $\alpha = 2.25$, the prevalence of the homogeneous case is higher only during one interval, with HET resulting on higher prevalence most of the time.

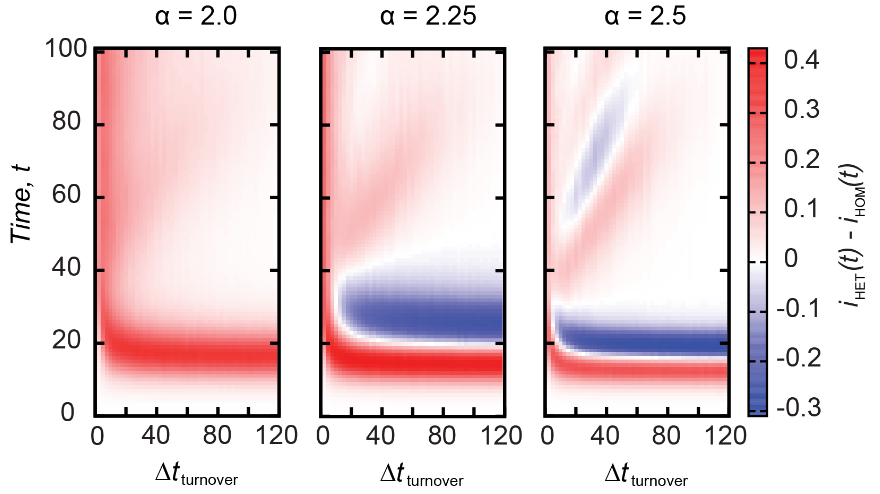

Figure S2: The difference in the prevalence of Δi for the heterogeneous and homogeneous contact patterns for different values of α. The x-axis corresponds to the turnover variable $\Delta t_{\text{turnover}}$ and the y-axis corresponds to the time steps.

4. Level of significance (p-value) for intensity and time of peak prevalence

To avoid excessive figures in the main text, we present here the p-value for the results of the intensity and time of peak prevalence. Figure S3A corresponds to results of Figure 3A, Figure S3B corresponds to results of Figure 3B, Figure S3C corresponds to results of Figure 4C and Figure S3D corresponds to results of Figure 4B. The blue regions correspond to statistically significant differences ($p < .01$) between the heterogeneous and homogeneous networks.

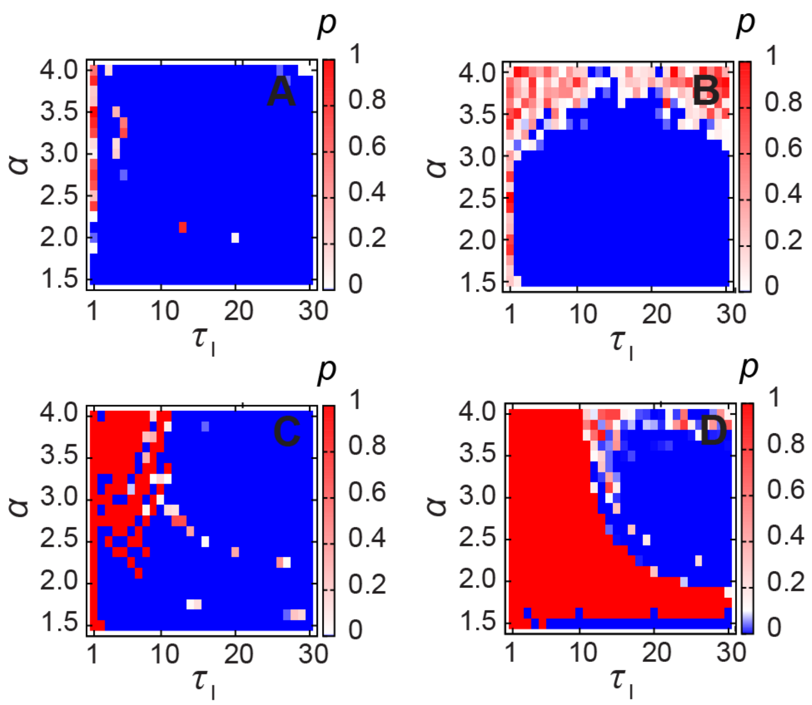

Figure S3: The panels give the level of significance (p-value) for: the intensity of the peak prevalence for (A) deterministic and (B) stochastic ($\lambda = 0.1$) SIR dynamics; the time of peak prevalence for (C) deterministic and (D) stochastic ($\lambda = 0.1$) SIR dynamics. The color blue means that $p < .01$.

5. Finite-size analysis of R_0 for SIR dynamics

We perform the simulations of the epidemics in the temporal network for different sizes N of the network to see if there is a dependence of R_0 and N. By using values of $N = \{1000, 2000, 4000, 8000, 16000\}$, we see that our estimation of R_0 is independent of the network size for SIR epidemics and is therefore a reliable estimate (Figure S4).

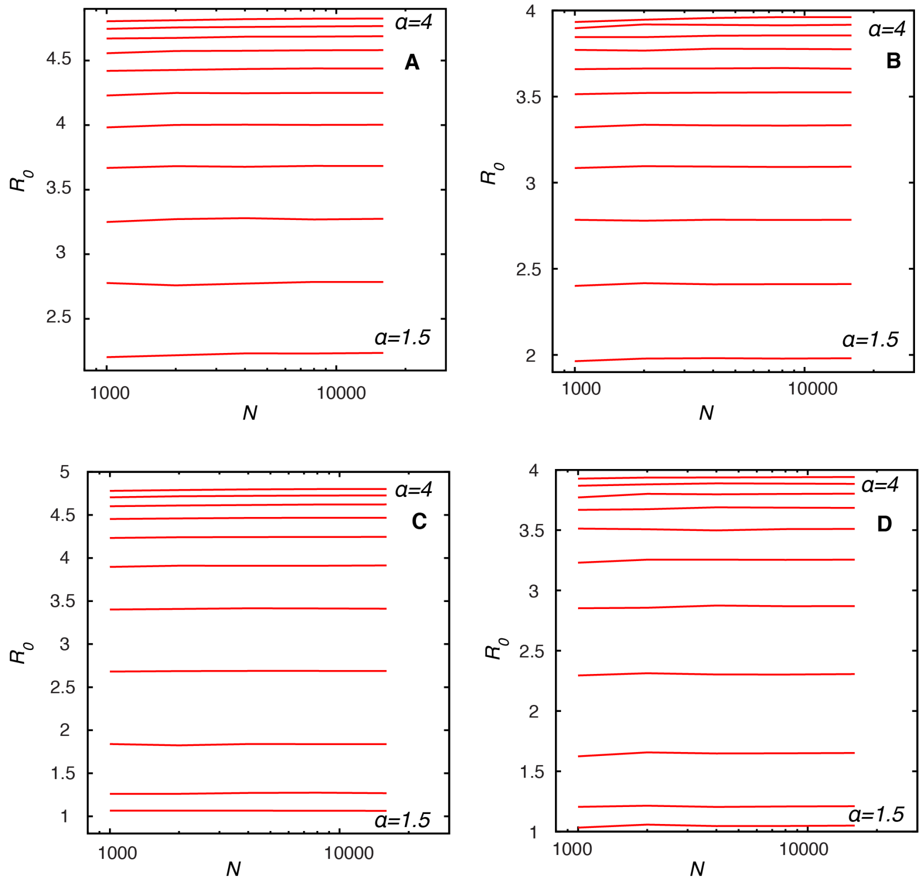

Figure S4: Estimation of R_0 for SIR epidemics for different sizes N of the temporal network. (a) SIR in heterogeneous network with $\Delta t_{\text{turnover}} \to \infty$; (b) SIR in homogeneous network with $\Delta t_{\text{turnover}} \to \infty$; (c) SIR in heterogeneous network with $\Delta t_{\text{turnover}} = 10$ and $\tau_I = 5$; (d) SIR in homogeneous network with $\Delta t_{\text{turnover}} = 10$ and $\tau_I = 5$. On each panel, from bottom to top, each curve corresponds to different growing values of α (at intervals of 0.25).

6. Epidemic outbreak for stochastic SIR dynamics

The number of infected and recovered vertices characterizes the outbreak size of an epidemic. In Figure S5 we show the outbreak $\langle \Theta_{\text{HET}}(t) \rangle$ at $t = 600$ (when the system is in the stationary state) for various values of the per-contact infection probability λ and infection duration τ_{I}. In Figure S5A,E, we set a threshold (white strip in the plot, corresponding to 0.1% of the population affected by the infection, i.e., $\langle \Theta_{\text{HET}}(t) \rangle = 0.001$) to illustrate the region where the epidemic occurs. This threshold closely overlaps with the threshold of $R_0 = 1$ in Figure 6A,E of the main text. In Figure S5B,F, we show the difference in the outbreak $\Delta\Theta$ for HET and HOM. We see the emergence of two patterns. The first, mostly around the bottom and the left of the plot (red region), corresponds to larger outbreaks for the HET contact patterns, and the other, in the upper right region (blue region), corresponds to larger outbreaks for the HOM contact patterns. In absolute values, when HET prevails it results in higher outbreaks than HOM. When $\Delta t_{\text{turnover}} = 10$, HET always result in larger outbreaks than HOM, and null outbreaks are observed for $\lambda < 0.2$ (Figure S5E).

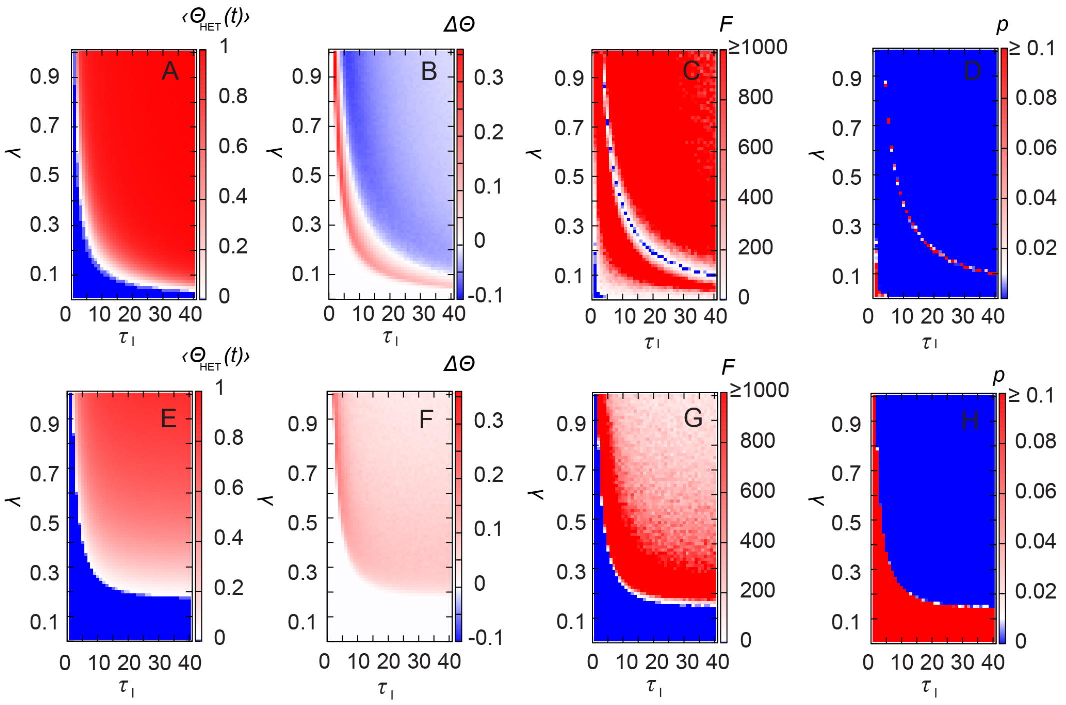

Figure S5: Numerical estimation of the outbreak size $\langle \Theta(t) \rangle$ (number of infected and recovered vertices) at $t = 600$ for HET network (with $\alpha = 2.5$) and the difference of

$\langle\Theta(t)\rangle$ between HET and HOM networks, that is, $\Delta\Theta = \langle\Theta_{\text{HET}}(t)\rangle - \langle\Theta_{\text{HOM}}(t)\rangle$. $\langle\Theta_{\text{HET}}(t)\rangle$ for HET in case of (A) $\Delta t_{\text{turnover}} \to \infty$ and (E) $\Delta t_{\text{turnover}} = 10$; $\Delta\Theta$ for (B) $\Delta t_{\text{turnover}} \to \infty$ and (F) $\Delta t_{\text{turnover}} = 10$; F statistics (red and white regions correspond to statistically significant differences, i.e. $F > F_c(1,98) = 6.901$, $p < .01$) for (C) $\Delta t_{\text{turnover}} \to \infty$ and (G) $\Delta t_{\text{turnover}} = 10$; raw p-values (blue means $p < .01$) for (D) $\Delta t_{\text{turnover}} \to \infty$ and (H) $\Delta t_{\text{turnover}} = 10$.

References

[S1] Clauset A, Shalizi CR, Newman MEJ (2009) Power law distributions in empirical data. *SIAM Review* 51(4): 661–703.
